# Nutritional status and metabolic alterations in patients with ataxia-telangiectasia

**DOI:** 10.1186/s13023-025-03785-2

**Published:** 2025-07-01

**Authors:** Cristiano do Amaral de Leon, Sérgio Luis Amantea, Renan Augusto Pereira, Ellen O. Dantas, Jéssica Loekmanwidjaja, Beatriz T. Costa-Carvalho, Juliana T. L. Mazzucchelli, Carolina S. Aranda, Maria E. González-Serrano, Elizabeth Alejandra De La Cruz Córdoba, Liliana Bezrodnik, Ileana Moreira, Janaira F. S. Ferreira, Vera M. Dantas, Valéria S. F. Sales, Carmen L. Navarrete, Maria M. S. Vilela, Isabella P. Motta, Jose Luis Franco, Julio Cesar Orrego Arango, Jesús A. Álvarez-Álvarez, Lina Rocío Riaño Cardozo, Julio C. Orellana, Antonio Condino-Neto, Cristina M. Kokron, Myrthes T. Barros, Lorena Regairaz, Diana Cabanillas, Carmen L. N. Suarez, Nelson A. Rosario, Herberto J. Chong-Neto, Olga A. Takano, Maria I. S. V. Nadaf, Lillian S. L. Moraes, Fabiola S. Tavares, Flaviane Rabelo, Jessica Pino, Wilmer C. Calderon, Daniel Mendoza-Quispe, Ekaterine S. Goudouris, Virginia Patiño, Cecilia Montenegro, Monica S. Souza, Aniela B. X. C. Castelo Branco, Wilma C. N. Forte, Flavia A. A. Carvalho, Gesmar Segundo, Marina F. A. Cheik, Pérsio Roxo-Junior, Maryanna Peres, Annie M. Oliveira, Arnaldo C. P. Neto, Maria Claudia Ortega-López, Alejandro Lozano, Natalia Andrea Lozano, Leticia H. Nieto, Anete S. Grumach, Daniele C. Costa, Nelma M. N. Antunes, Victor Nudelman, Camila T. M. Pereira, Maria D. M. Martinez, Francisco J. R. Quiroz, Aristoteles A. Cardona, Maria E. Nuñez-Nuñez, Jairo A. Rodriguez, Célia M. Cuellar, Gustavo Vijoditz, Daniélli C. Bichuetti-Silva, Carolina C. M. Prando

**Affiliations:** 1 Graduate Program in Medical Sciences, Universidade Federal de Ciências da Saúde de Porto Alegre (UFCSPA), Rua Sarmento Leite, 245, Centro Histórico, Porto Alegre, RS 90050-170 Brazil; 2 Pediatrics Department, UFCSPA, Rua Sarmento Leite, 245, Centro Histórico, Porto Alegre, RS 90050-170 Brazil; 3 Pediatrics Department, Universidade Federal de São Paulo (UNIFESP), Rua Botucatu, 598, Vila Clementino, São Paulo, SP 04023-062 Brazil; 4 Universidade Federal de São Paulo, Rua Sena Madureira, 1500, Vila Clementino, São Paulo, SP 04021-001 Brazil; 5 Instituto Nacional de Pediatria, Avenida Insurgentes Sur, 3700-Letra C, Insurgentes Cuicuilco, Coyoacán, 04530 Ciudad de México, México Ciudad de México Mexico; 6 Hospital de Niños Ricardo Gutierrez, Gallo 1330, C1425EFD Ciudad Autónoma de, Buenos Aires, Argentina; 7 Hospital Infantil Albert Sabin, Rua Tertuliano Sales, 544, Vila União, Fortaleza, CE 60410-794 Brazil; 8 Universidade Federal do Rio Grande do Norte (UFRN), Avenida Senador Salgado Filho, 3000, Campus Universitário, Lagoa Nova, RN 59078-970 Brazil; 9 Inmune Centro De Vacunas, 100129 Ciudad del Este, Alto Paraná Paraguay; 10 Universidade Estadual de Campinas (UNICAMP), Avenida Albert Einstein, 901, Cidade Universitária “Zeferino Vaz”, Barão Geraldo, Campinas, SP 13083-852 Brazil; 11 Universidad de Antioquia, UdeA, Cl. 67 #53–108, Medellin, Antioquia Colombia; 12 Hospital de Niños de la Santisima Trinidad, Bajada Pucará 787, X5000 Cordoba, Argentina; 13 Hospital das Clínicas FMUSP, Universidade de São Paulo, Rua Doutor Ovídio Pires de Campos, 225, Cerqueira César, São Paulo, SP 05403-010 Brazil; 14 Hospital de Niños Sor Maria Ludovica, Calle 14 1631, La Plata, Buenos Aires, Argentina; 15 Hospital Roberto del Rio, Profesor Zañartu, 1085, Independencia, Santiago, Chile; 16 Universidade Federal do Paraná (UFPR), Rua XV de Novembro, 1299, Centro, Curitiba, PR 80060-000 Brazil; 17 Universidade Federal do Mato Grosso, Rua Quarenta e Nove, 2367, Boa Esperança, Cuiabá, MT 78060-900 Brazil; 18 Hospital da Criança de Brasília José de Alencar, AENW 3, Lote A, Setor Noroeste, Brasília, DF 70684-831 Brazil; 19 Fundación Valle del Lili Hospital, Cra. 98 #18–49, Comuna 17, Cale, Valle del Cauca, Colombia; 20 Faculty of Medicine, Universidad Nacional Mayor de San Marcos (UNMSM), Av. Carlos Germán Amezaga #375, Lima, Peru; 21 Universidade Federal do Rio de Janeiro, Rua Antônio Barros de Castro, 119, Cidade Universitária, Rio De Janeiro, RJ 21941-853 Brazil; 22 Hospital de Pediatría del Centro Hospitalario Pereira Rossell, Bulevar Artigas 1590, Lord Ponsoby 2410, 11600 Montevideo, Uruguay; 23 Hospital Federal dos Servidores do Estado, Rua Sacadura Cabral, 178, Saúde, Rio de Janeiro, RJ 20221-161 Brazil; 24 Faculdade de Ciências Médicas da Santa Casa de São Paulo, R. Jaguaribe, 155, Vila Buarque, São Paulo, SP 01224-001 Brazil; 25 Instituto Nacional de Saúde da Mulher, da Criança e do Adolescente Fernandes Figueira (IFF/Fiocruz), , Avenida Rui Barbosa, 716, Flamengo, Rio de Janeiro, RJ 22250-020 Brazil; 26 Universidade Federal de Uberlândia (UFU), Avenida João Naves de Ávila, 2121, Santa Mônica, Uberlândia, MG 38408-100 Brazil; 27 Division of Immunology and Allergy, Department of Pediatrics, Ribeirão Preto Medical School, Universidade de São Paulo, Avenida Bandeirantes, 3900, Ribeirão Preto, SP 14048-900 Brazil; 28 Hospital Universitário Presidente Dutra, Rua Barão de Itapari, 227, Centro, São Luís, MA 65020-070 Brazil; 29 Universidade de Passo Fundo (UPF), BR 285 km 292,7, Campus I, Bairro São José, São José, Passo Fundo, RS 99052-900 Brazil; 30 Hospital Universitário Infantil de San José, Cra. 52 #67a-71, Barrios Unidos, Bogotá, Cundinamarca Colombia; 31 Universidad Católica de Córdoba, Avenida Armada Argentina 3555, X5017 Córdoba, Argentina; 32 Hospital del Niño DIF Hidalgo, Blvd. Felipe Ángeles, Km 84.5, Venta Prieta, 42083 Pachuca de Soto, Hgo., Pachuca de Soto, Mexico; 33 Centro Universitário Faculdade de Medicina do ABC (FMABC), Avenida Lauro Gomes, 2000, Vila Sacadura Cabral, Santo André, SP 09060-870 Brazil; 34 Hospital de Clínicas de Passo Fundo, RuaTiradentes, 295, Centro, Passo Fundo, RS 99010-260 Brazil; 35 Universidade Estadual de Montes Claros, Avenida Professor Rui Braga, s/n, Vila Mauriceia, Montes Claros, MG 39401-089 Brazil; 36 Hospital Israelita Albert Einstein, Avenida Albert Einstein, 627/701, Morumbi, São Paulo, SP 05652-900 Brazil; 37 Division of Allergy Clinical Immunology and Rheumatology, Universidade Federal de São Paulo (UNIFESP), Rua dos Otonis 725, Vila Clementino, São Paulo, SP 04025-002 Brazil; 38 Centro Medico Nacional La Raza (CMN Raza), P.º de las Jacarandas S/N, La Raza, Azcapotzalco, 02990 Ciudad de México, CDMX Mexico; 39 Instituto Hondureño de Seguridad Social, GXRM + QQ9, San Pedro Sula, 21102 Honduras; 40 Universidad Autónoma de Aguascalientes, Avenida Universidad # 940, Ciudad Universitaria, C.P. 20100 Aguascalientes, Ags. México; 41 Hospital Civil de Guadalajara Dr Juan I Menchaca, Salvador Quevedo y Zubieta, 750, Independencia Oriente, 44340 Guadalajara, , Jal. Mexico; 42 Universidad Surcolombiana, Avenida Pastrana Borrero, Carrera 1, Neiva, Huila, Colombia; 43 Instituto de Medicina Tropical, PCC4 + RPC, 001535 Asunción, Paraguay; 44 Hospital Nacional Alejandro Posadas, Avenida Presidente Arturo U. Illia s/n y Marconi Morón 386, B1684 El Palomar, Provincia de Buenos Aires Argentina; 45 Universidade Federal de Goiás (UFG), Av. Esperança, s/n - Chácaras de Recreio Samambaia, Goiânia, GO 74690-900 Brazil; 46 Hospital Pequeno Príncipe, Rua Desembargador Motta, 1070, Água Verde, Curitiba, PR 80250-060 Brazil

**Keywords:** Ataxia telangiectasia syndrome, Malnutrition, Lipid metabolism disorders, Glucose metabolism disorders, Aminotransferases

## Abstract

**Background:**

Ataxia-telangiectasia (A-T) is a DNA repair disorder characterized by progressive degeneration, immunodeficiency, cancer predisposition, malnutrition, metabolic disorders, and chronic liver disease. The study aims to describe the nutritional status and plasma levels of biomarkers of lipid status, metabolic profile, and liver function of patients with A-T.

**Results:**

A total of 218 patients from 9 Latin American countries were included in the study. The distribution of patients according to nutritional status by age group revealed an over-time increase in the proportion of patients with severe thinness (*p* = 0.016). High glucose and triglyceride levels were observed in 9.5% and 23.6% of patients, respectively. Total cholesterol was high in 31.7, and 34.0% had abnormal LDL-c levels. In the analysis of paired samples, a progressive increase in aspartate aminotransferase was observed over time.

**Conclusions:**

The present results are comparable to those of previous studies also showing changes in nutritional status and in lipid, metabolic, and liver profiles over time. These findings confirm a high rate of thinness in patients with A-T and progressive deterioration as the disease progresses, as well as changes in plasma levels of biomarkers of lipid status, metabolic profile, and liver function.

## Background

Ataxia-telangiectasia (A-T) or Louis-Bar Syndrome (ORPHA:100, OMIM #208,900) is a rare, inherited form of autosomal recessive neurodegenerative ataxia with onset in early childhood [1–3]. According to the 2022 classification of inborn errors of immunity, patients with A-T are included in the group of genetic syndromes associated with immune abnormalities [4]. A-T has a variable incidence of 1:40,000 to 1:100,000, with both sexes affected equally [5]. It is caused by mutations in the ATM (Ataxia-Telangiectasia, Mutated) gene located on chromosome 11q22-23, whose translated protein (ATM) belongs to the phosphatidylinositol 3-kinase family and is involved in mitogenic signal transduction, intracellular protein transport, and regulation of the DNA repair machinery [6]. Failure of these processes results in chromosomal instability, increased production of reactive oxygen species (ROS), mitochondrial dysfunction with concomitant oxidative stress, cell cycle arrest, and cell apoptosis.

A-T is characterized by progressive neurodegeneration, oculocutaneous telangiectasia, increased susceptibility to infections, radiosensitivity, cancer predisposition, malnutrition, metabolic and cardiovascular disorders, dysphagia, and more recently described, chronic liver disease [7–9]. Clinical and biochemical changes that have been associated with the classic A-T phenotype include reduced lean body mass, premature aging, insulin resistance, type 2 diabetes, and increased risk of developing cardiovascular disease [10].

Liver disease associated with A-T typically appears during the second decade of life, with more than 90% of older individuals showing liver abnormalities [11, 12]. Liver involvement belongs to the complex premature aging component of the syndrome, which also includes insulin resistance, type 2 diabetes, and dyslipidemia, thus causing incomplete metabolic syndrome [13, 14]. However, hepatopathy is usually mild and does not limit the synthesis and detoxification functions of the liver [11].

A-T is classified as classic or atypical depending on the homozygous or heterozygous mutation in the ATM gene, respectively. The clinical phenotype and severity of A-T depend on the presence of residual ATM kinase activity as determined by the genotype [15]. Mutations that result in an almost complete loss of functional ATM protein kinase lead to the classic form of the disease. Residual ATM kinase activity can reduce oxidative stress to maintain mitochondrial function or to compensate for the absence of functional ATM protein (eg, gene modification and environmental factors), which may be responsible for the mild clinical manifestations of atypical cases of A-T [16].

Patients with classic A-T tend to be shorter and often experience lower-than-expected height and weight gain, which can start during the intrauterine period and persist throughout life. Even if the child appears to have a good growth rate during the first 2 years of life, the poor weight and height gain starts early and becomes persistent [17]. Malnutrition in children is particularly worrying because it negatively affects normal height and weight gain and may have a detrimental impact on lung development. Cross-sectional studies have shown that patients with A-T have high rates of malnutrition, short stature, and reduced lean body mass [18, 19]. In addition, changes in nutritional status increase infection-related morbidity and mortality [20]. Children, adolescents and young adults with A-T have a high mortality rate, with an average age at death of 14 years due to various complications such as neoplasms, infections, and nutritional complications, among others [21].

Managing A-T poses significant challenges because of the disease’s complexity. Current treatments primarily focus on alleviating symptoms as effectively as possible. Recent studies have explored therapies such as N-acetyl-DL-leucine for ataxia symptoms, bone marrow transplant therapy to restore immune competence, and gene therapy to correct defective alleles in the ATM gene—potentially restoring ATM protein function and reducing DNA damage. Additionally, the use of dexamethasone in modified erythrocytes (EryDex) and growth hormone have shown promising results, demonstrating improvements in locomotor behavior and immunity with few adverse effects [22].

Assessing malnutrition and biomarkers of metabolic syndrome is essential for the clinical management of A-T. However, there is a paucity of literature on the topic mainly due to the rarity of the disease. Epidemiological research is needed to expand knowledge of the natural history and improve the care of individuals with A-T in daily practice. This study aimed to describe the nutritional status and plasma levels of biomarkers of lipid status, metabolic profile, and liver function of Latin American patients with A-T.

## Methods

All referral centers (n = 111) participating in the Latin American Society for Immunodeficiencies registry (https://registrolasid.org/docs/estatisticas_lasid-2016_jun.pdf) were formally invited by email to participate. Information from the participating centers was reviewed retrospectively and the data were entered in a semi-structured survey through an online questionnaire by the principal investigator. The following data were analyzed from medical records: weight, height, fasting glucose, total cholesterol, low-density lipoprotein cholesterol (LDL-c), triglycerides, alpha-fetoprotein (AFP), aspartate aminotransferase (AST), and alanine transaminase (ALT).

Age- and sex-specific body mass index (BMI) z-scores were calculated using the World Health Organization criteria to assess nutritional status in individuals aged 2 to 20 years (severe thinness if z-score < − 3; thinness if z-score between − 3 and − 2; normal weight if z-score between − 2 and + 1) [23, 24]. Cases where the z-score was > + 1 were grouped together as patients at risk of overweight, obesity, or severe obesity.

Abnormal total cholesterol levels were ≥ 170 mg/dL for children/adolescents and ≥ 200 mg/dL for adults, abnormal LDL-c levels were ≥ 110 mg/dL for children/adolescents and > 129 mg/dL for adults, and abnormal triglyceride levels were ≥ 100 mg/dL for children/adolescents and ≥ 150 mg/dL for adults [25, 26]. Blood glucose was considered abnormal if serum levels were ≥ 100 mg/dL. AST was considered abnormal if ≥ 60 (U/L) in children/adolescents, ≥ 40 (U/L) in adult men, and ≥ 32 (U/L) in adult women. ALT was considered abnormal if ≥ 40 (U/L). AFP was considered abnormal if > 20 ng/mL [27].

Upon analyzing the medical records, we observed that both the first and last patient assessments were duly documented, starting from the initial consultation at their respective health centers through the end of data collection in 2018. However, we could not accurately determine the exact time interval between these assessments due to multiple biomarker collections performed for each patient over the study period. Therefore, to analyze the biomarker trends throughout the study, we decided to compare each variable based on the first and last records available in the medical records.

This research is part of a recently published larger study analyzing the metabolic, immunological, and mortality profile of patients with A-T in Latin America [21]. For the present analysis, we adopted the classification into 3 categories, according to the European Society for Immunodeficiencies (ESID) criteria [28], for the diagnosis of A-T and composition of our sample, as follows: *Definitive* diagnosis: defined as the presence of pathogenic variants in both alleles of the ATM gene and either increased radiation-induced chromosomal breakage in cultured cells or progressive cerebellar ataxia; *Probable* diagnosis: defined as the presence of progressive cerebellar ataxia and at least 3 of the following 4 abnormalities – (1) ocular or facial telangiectasia, (2) AFP at least 2 SD above normal for age, (3) serum IgA at least 2 SD below normal for age, or (4) increased radiation-induced chromosomal breakage; and *Possible* diagnosis: defined as the presence of progressive cerebellar ataxia and at least 1 of the 4 abnormalities described above.

The data were analyzed descriptively and expressed as absolute and relative frequencies. Associations between variables were examined using the chi-square test or Fisher’s exact test. If statistical differences occurred in the distributions, standardized adjusted residual analysis was used to identify local differences, where absolute values above 1.96 indicated evidence of (local) associations between the relative categories. Percentages at 2 or 3 assessment points (related samples) were compared using the McNemar test and Cochran Q test, respectively.

This study was approved by the research ethics committees of the participating centers (CAAE: 61,257,916.3.1001.5505) and conducted in accordance with the provisions of the Declaration of Helsinki. Furthermore, informed consent for data collection and use was obtained from each patient and/or family member.

## Results

Data from 218 patients with A-T from 46 health centers located in 9 Latin American countries were collected between July 2015 and February 2018. These patients had a mean age of 13.7 years at the time of the survey. According to the ESID criteria, 90 patients (41.5%) had a *probable* A-T diagnosis, 59 patients (27.2%) had a *possible* A-T diagnosis, and 69 patients (31.8%) had a *definitive* A-T diagnosis. Overall, 123 patients were from Brazil, 34 were from Mexico, 32 were from Argentina, 13 were from Colombia, 5 were from Chile, 4 were from Peru, 3 were from Uruguay, 3 were from Paraguay, and 1 was from Honduras. A total of 111 patients (51.0%) were female and 107 (49.0%) were male. Ataxia occurred in all patients and was the initial presenting symptom in 159 patients (72.9%), dysarthria in 156 patients (85.2%), postural changes in 144 (75.4%), and ocular apraxia in 133 (74.7%); 112 patients used a wheelchair (56.3%).

Higher proportions of severe thinness were observed among older individuals: 9.1% in patients aged 2 to 6 years; 19.0% in patients aged 7 to 10 years; 39.1% in patients aged 11 to 19 years; and 50.0% in patients aged ≥ 20 years. The distribution of patients according to nutritional status by age group is shown in Table 1.

**Table 1:** Patients’ distribution according to nutritional status by age group

Nutritional status by age group	N	%
2 to 6 years	77	100.0
Severe thinness	7	9.1
Thinness	7	9.1
Normal weight	60	77.9
Risk of overweight/obesity/severe obesity	3	3.9
Missing data	141	
7 to 10 years	100	100.0
Severe thinness	19	19.0
Thinness	24	24.0
Normal weight	49	49.0
Risk of overweight/obesity/severe obesity	8	8.0
Missing data	118	
11 to 19 years	69	100.0
Severe thinness	27	39.1
Thinness	9	13.0
Normal weight	28	40.6
Risk of overweight/obesity/severe obesity	5	7.2
Missing data	149	
Adults	8	100.0
Severe thinness	4	50.0
Thinness	0	0.0
Normal weight	3	37.5
Risk of overweight/obesity/severe obesity	1	12.5
Missing data	210	

Patients’ gastrointestinal clinical features are shown in Table 2.

**Table 2:** Patients’ gastrointestinal clinical features

Clinical feature	N	%
Dysphagia	196	100.0
Yes	98	50.0
No	98	50.0
Hepatic steatosis	168	100.0
Yes	24	14.3
No	144	85.7
Gastrostomy	193	100.0
Yes	16	8.3
No	177	91.7

Data from patients who had more than one anthropometric assessment over time were paired to assess the over-time evolution of the z-scores individually. The proportion of patients with A-T with severe thinness increased with increasing age (*p* = 0.016) (Table 3).

**Table 3:** Patients’ distribution according to nutritional status: paired sample

Variable	Age group					
	2 to 6 years		7 to 10 years		11 to 19 years	
	N	%	N	%	N	%
Nutritional status	18	100.0	18	100.0	18	100.0
Severe thinness	3	16.7	3	16.7	8	44.4
Thinness	2	11.1	4	22.2	3	16.7
Normal weight	13	72.2	9	50.0	7	38.9
Risk of overweight, obesity, severe obesity	0	0.0	2	11.1	0	0.0

Cochran Q test for severe thinness: *p* = 0.016

Table 4 shows the results of the lipid profile, fasting glucose, liver enzymes, and AFP of the study sample. In patients with more than one AST and ALT measurement, paired analyses showed a tendency for liver enzymes to increase over time (Tables 5 and 6).

**Table 4:** Laboratory results

Variable	N	%
Alpha-fetoprotein (ng/mL)	194	100.0
Normal	11	5.7
High	183	94.3
Not assessed	24	
Blood glucose (mg/dL)	158	100.0
Normal	143	90.5
High	15	9.5
Not assessed	60	
Triglycerides (mg/dL)	123	100.0
Normal	94	76.4
High	29	23.6
Not assessed	95	
Total cholesterol (mg/dL)	126	100.0
Normal	86	68.3
High	40	31.7
Not assessed	92	
LDL cholesterol (mg/dL)	116	100.0
Normal	82	70.7
High	34	29.3
Not assessed	102	

*LDL* Low-density lipoprotein

**Table 5:** Patients’ distribution by AST and ALT

Variable	First assessment		Last assessment	
	N	%	N	%
AST (U/L)	161	100.0	61	100.0
High	35	21.7	32	52.5
Normal	126	78.3	29	47.5
ALT (U/L)	160	100.0	61	100.0
High	53	33.1	36	59.0
Normal	107	66.9	25	41.0

*ALT* Alanine transaminase; *AST* Aspartate aminotransferase

**Table 6:** Patients’ distribution by AST and ALT: paired sample

Variable	First assessment		Last assessment		p*
	N	%	N	%	
AST (U/L)	61	100.0	61	100.0	< 0.001
High	12	19.7	32	52.5	
Normal	49	80.3	29	47.5	
ALT (U/L)	61	100.0	61	100.0	0.019
High	25	41.0	36	59.0	
Normal	36	59.0	25	41.0	

*ALT* Alanine transaminase; *AST* Aspartate aminotransferase

^*^ McNemar test

## Discussion

This multicenter study evaluated 218 patients with A-T in Latin America and showed a high rate of thinness, changes in hepatic inflammatory markers, atherogenic lipid profile, and glucose abnormalities in this population, as well as a significant decline in BMI z-scores over time. This is consistent with the findings of Krauthammer et al., who reported a drop in BMI z-scores over time despite regular follow-up in a multidisciplinary A-T center, where 61.4% of patients had severe thinness and 22.7% had thinness during the follow-up period [29]. Stewart et al. also described a significant decline in z-scores despite optimal nutritional therapy, where approximately 25% of patients were classified as thin (z-score < − 2) at some point during the study [30].

Problems with eating, swallowing and nutrition are common in patients with A-T. In our series, 50% of patients had dysphagia and 8.3% had undergone gastrostomy. Gastrostomy has been associated with an improvement in nutritional status or at least a decline in the progression of malnutrition, suggesting that malnutrition is not an inevitable consequence of A-T but rather a complication that can be successfully managed with early intervention [30]. However, the benefits of gastrostomy are still controversial in the literature. Natale et al. showed no benefit of gastrostomy for nutritional status, indicating its use only in very severe patients [17]. Previous reviews have highlighted the importance of preventing secondary complications of dysphagia, where gastrostomy feeding may be required to prevent pulmonary and nutritional complications [15].

Our study also assessed hepatic inflammatory markers, showing that both AST and ALT changed over time in patients with A-T. Oxidative stress has been recently linked to increased inflammatory response leading to liver damage. Harbort et al. demonstrated that neutrophils from patients with A-T produce significantly more cytokines and live longer than controls, suggesting that innate immune dysfunction may drive liver inflammation in A-T [31]. Oxidative stress is a key mechanism in the pathogenesis of A-T, contributing particularly to neurodegeneration and morbidities such as liver disorders and dyslipidemia [9, 32].

Paulino et al. found elevated levels of AST and ALT in adolescent patients with A-T [9]. In a retrospective study, Donath et al. reported a progressive increase in ALT and gamma-glutamyltransferase (GGT) especially after 12 years of age [12]. Also in a retrospective series, Weiss et al. showed an increase in liver enzymes in young patients with A-T at a mean (SD) age of 9.97 (5.09) years, as well as a significant association with dyslipidemia [11]. Barreto et al. demonstrated a correlation between liver biomarkers (ALT, AST, and GGT) as predictors of liver fibrosis when their values were greater than or equal to twice the upper limit of normal, with a sensitivity of 80% and specificity of 95% [33]. Therefore, serial monitoring of AST and ALT levels from the age of 10 years is highly recommended to screen for liver fibrosis in A-T. Andrade et al. found higher levels of ALT, LDL-c, and triglycerides in thin patients, linking them to liver dysfunction and insulin resistance [34]. The clinical significance of liver disease in A-T is currently unclear, but more than 90% of patients develop elevated liver enzymes with advancing age, which is associated with a high degree of hepatic steatosis and occasionally liver tissue fibrosis [12].

AFP is a glycoprotein produced in large amounts by the fetal liver during normal development that is involved in the transport of different ligands, chemotaxis, free radical scavenging, and lipid peroxidation [35]. AFP production decreases after birth and reaches a plateau (adult values) at around 2 years of age [35]. AFP is mainly known as a marker for hepatocellular carcinoma, but it can be elevated in germ cell tumors, viral hepatitis, liver fibrosis, and neurodegenerative diseases such as A-T [35]. The elevation of AFP is a biological characteristic of patients with A-T and easy to detect early [36]. Our study detected AFP alterations in 94.3% of patients, consistent with data from the literature showing that this marker is useful in clinical practice when the disease is suspected. Also, 14.3% of the patients in our series had hepatic steatosis, supporting the findings of Donath et al., who demonstrated a significant correlation between AFP and hepatic steatosis [37].

Our study also confirmed the atherosclerotic lipid profile previously described in patients with A-T. We found an increase in triglycerides in 23.6% of patients, total cholesterol in 31.7%, and LDL-c in 29.3%. Andrade et al. highlighted the risk of cardiovascular disease by showing an atherogenic lipid profile and presence of hepatic steatosis in 64.7% of patients with A-T [38]. Similarly, Weiss et al. showed an association between A-T and dyslipidemia [11]. In an experimental study, Mercer et al. showed that ATM protein deficiency accelerates the atherosclerotic process and promotes mitochondrial DNA damage, which can lead to increased ROS production and reduced oxidative phosphorylation that are directly related to changes in lipid and glucose metabolism in A-T [39].

Diabetes mellitus is associated with insulin resistance, hyperglycemia, inflammation, mitochondrial dysfunction, endothelial dysfunction, and oxidative stress [40]. Insulin resistance and diabetes mellitus can occur especially later in the course of A-T [15]. A study of mice with a heterozygous ATM mutation and apolipoprotein E deficiency, involved in the redistribution of triglycerides and cholesterol in different tissues, showed accelerated progression of atherosclerotic lesions in association with insulin resistance and glucose intolerance [41]. In our sample, 9.5% of patients had elevated fasting glucose. In a recent publication, Riboldi et al. corroborated the findings of the occurrence of insulin resistance, diabetes mellitus, hypercholesterolemia, and hepatic steatosis especially in more advanced stages of the disease [15]. Diabetes mellitus has recently been recognized as a common finding in older patients with A-T, often beginning at puberty, and an annual diabetes screening is therefore recommended for patients over 12 years of age [14].

Scientific research are underway to test the efficacy of different treatment approaches for A-T. Leuzzi et al. have pioneered a treatment using erythrocyte-delivered dexamethasone (EryDex), which has demonstrated improvements in neurological symptoms while avoiding the typical side effects associated with steroids [42]. Additionally, Saberi-Karimian et al. reported success treating a 6-year-old patient with a regimen combining physical therapy, vitamin E supplementation, and oral dexamethasone at a dosage of 0.075 mg/kg/day. After 28 days, the patient’s scale for the assessment and rating of ataxia (SARA) score showed a significant improvement, accompanied by notable gains in handwriting and painting abilities [43].

N-acetyl-DL-leucine, a compound used since 1957 for acute vertigo and central vestibular compensation, has shown positive effects on ataxia symptoms in patients with A-T, without significant side effects. Saberi-Karimian et al. reported positive outcomes in a 9-year-old patient treated with N-acetyl-DL-leucine over 16 weeks, with a dosage adjusted to 4 g/day (2 g in the morning and 2 g in the evening). The treatment led to a 48.88% improvement in the SARA score, along with the resolution of nausea and constipation, and weight gain, without any adverse drug effects [44]. A recent randomized clinical trial investigating the efficacy of N-acetyl-L-leucine, the active enantiomer of N-acetyl-DL-leucine, in patients with A-T showed that while it improved patients’ nausea and constipation, it did not significantly benefit ataxia symptoms, biochemical parameters, or food intake. Despite the lack of substantial changes in ataxia symptoms in this trial, the results suggest that N-acetyl-L-leucine may still serve as a potential adjuvant therapy for managing gastrointestinal symptoms in patients with A-T [45].

The present study describes a considerable number of patients with A-T in Latin America, providing relevant information on malnutrition, hepatic inflammatory markers, and atherogenic lipid and glucose profiles in ethnically diverse patients from 9 Latin American countries. Significant samples of rare diseases are not easy to obtain. Nevertheless, this study has limitations. Firstly, the retrospective cross-sectional design of the study, based on questionnaires, may introduce selection and information biases. Secondly, longitudinal data may be limited in the case of loss to clinical follow-up. Thirdly, the clinical spectrum of A-T is not uniform; different ATM mutations, residual ATM protein function and level of ATM kinase activity may lead to a variety of clinical and laboratory phenotypes [46].

## Conclusions

This multicenter study of 218 Latin American patients with A-T showed a high rate of thinness, changes in hepatic inflammatory markers, atherogenic lipid profile, and glucose abnormalities in this population, as well as a significant decline in BMI z-scores over time. The hepatic inflammatory markers AST and ALT changed over time, indicating an atherosclerotic lipid profile. The need to prevent malnutrition should be considered to improve patient quality of life, whether by optimal nutritional therapy, follow-up with a qualified health care professional, or invasive interventions such as gastrostomy tube placement.

## Data Availability

The datasets used and/or analysed during the current study are available from the corresponding author on reasonable request.

## Abbreviations

AFP: Triglycerides, α-fetoprotein
ALT: Alanine transaminase
AST: Aspartate aminotransferase
A-T: Ataxia-telangiectasia
ATM: Ataxia-telangiectasia, mutated
BMI: Body mass index
GGT: Gamma-glutamyltransferase
LDL-c: Low-density lipoprotein cholesterol
SARA: Scale for the assessment and rating of ataxia
